# Chronic exposure to cigarette smoke extract increases nicotine withdrawal symptoms in adult and adolescent male rats

**DOI:** 10.3389/adar.2023.11324

**Published:** 2023-09-07

**Authors:** Daisy D. Reynaga, Michelle Cano, James D. Belluzzi, Frances M. Leslie

**Affiliations:** Department of Pharmaceutical Sciences, University of California, Irvine, Irvine, CA, United States

**Keywords:** dependence, adolescent, nicotine, cigarette smoke, tobacco constituents

## Abstract

The aim of the current study was to determine whether non-nicotine constituents of cigarette smoke contribute to nicotine dependence in adolescent and adult male Sprague Dawley rats. For 10 days animals were given three times daily intravenous injections of nicotine (1.5 mg/kg/day) or cigarette smoke extract (CSE) containing an equivalent dose of nicotine. Both spontaneous and mecamylamine-precipitated withdrawal were then measured. Chronic treatment with CSE induced significantly greater somatic and affective withdrawal signs than nicotine in both adolescents and adults. Mecamylamine-precipitated somatic signs were similar at both ages. In contrast, animals spontaneously withdrawn from chronic drug treatment exhibited significant age differences: whereas adolescents chronically treated with nicotine did not show somatic signs, those treated with CSE showed similar physical withdrawal to those of adults. Mecamylamine did not precipitate anxiety-like behavior at either age. However, both adolescents and adults showed significant anxiety in a light-dark box test 18 h after spontaneous withdrawal. Anxiety-like behavior was still evident in an open field test 1 month after termination of drug treatment, with adolescents showing significantly greater affective symptoms than adults. Our findings indicate that non-nicotine constituents of cigarette smoke do contribute to dependence in both adolescents and adults and emphasize the importance of including smoke constituents with nicotine in animal models of tobacco dependence.

## Introduction

Physiological dependence is a key aspect of compulsive drug use [1]. Smoking cessation can result in both somatic withdrawal signs, including insomnia and bradycardia, and affective signs such as craving, restlessness, irritability, anxiety, and difficulty concentrating [2]. These withdrawal signs can emerge within 20 min after cessation and may persist for months [2, 3].

Most tobacco use begins during adolescence [3]. Whereas teen smoking has declined in the United States within the last decade, the use of electronic cigarettes (e-cigarettes) has escalated [4–6]. Clinical studies report that teens are sensitive to withdrawal from cigarette use, showing signs of dependence soon after initiation and before establishing daily use [7, 8]. Teens can also become addicted to e-cigarettes with dependence correlating highly with salivary cotinine, a nicotine metabolite [6, 9, 10]. Although there are limited data on withdrawal from e-cigarettes, there are likely several common features between cigarette and e-cigarette withdrawal, such as craving, tolerance, and impaired control [11]. In one study of adults, ∼25% of e-cigarette users had withdrawal symptoms upon quitting which were less severe than that caused by quitting tobacco cigarettes [12]. A recent study of adolescents suggests similar levels of dependence in users of e-cigarettes and combustible cigarettes [13].

Animal studies have shown that nicotine withdrawal symptoms in adult rodents are similar to those of humans smokers [14–16]. However, there are substantial differences in the findings of clinical and preclinical studies of withdrawal in adolescents. Whereas teenagers are very sensitive to the effects of smoking cessation [7, 8], rodent adolescents show fewer somatic and affective signs of nicotine withdrawal than adults [17–19]. One possible reason for this discrepancy is that most animal studies use nicotine alone without non-nicotine constituents found in cigarette smoke. Studies in which tobacco smoke is chronically administered via inhalation induces physical dependence in both adult and pre-adolescent rats [20–22]. Others have shown that inhibition of monoamine oxidase, as occurs with tobacco smoke [23, 24], potentiates nicotine withdrawal [25, 26]. However, none of these studies have done systematic comparisons across drugs and ages, as our current study does.

Use of aqueous cigarette smoke extract (CSE) permits the study of the collective effects of thousands of cigarette smoke constituents on withdrawal. In solution form it allows control of daily drug exposure, which may not be possible via inhalational exposure. We have previously found that CSE self-administration is associated with increased stress-induced reinstatement as compared to nicotine alone [24, 27], which may indicate that tobacco constituents sensitize animals to stress responses and to the negative effects of tobacco dependence. The present study tests this hypothesis by comparing the effects of daily exposure to CSE and nicotine on both somatic and affective signs of spontaneous and precipitated withdrawal in adolescent and adult male rats. This work is part of the dissertation of the first author [28].

## Materials and methods

The materials and methods used in this study have been described previously [28].

### Animals

Male Sprague Dawley rats (Charles River Labs, Hollister CA) were delivered at postnatal day (P)17 with dam or at P81 and were housed 2–3 per cage in a humidity- and temperature-controlled vivarium with a 12 h light cycle, with lights turned on at 7 a.m. daily. Pups were weaned at P21 and similarly housed. To reduce surgical stress, animals were handled for 2 days prior to catheterization surgery. Adolescents were free-fed while adults were food restricted to be kept at a 95% free-feeding weight during the duration of experiments. All experimental procedures followed NIH guidelines and were approved by the Institutional Animal Care and Use Committee of the University of California, Irvine.

### Drugs

Nicotine hydrogen tartrate (Sigma, St. Louis, MO) was dissolved in sterile saline and adjusted to pH 7.2–7.4, with dose calculated as free base. Mecamylamine HCl (Tocris Bioscience, Bristol, United Kingdom) was dissolved in sterile saline with dose calculated as salt. CSE was produced by bubbling commercial cigarette smoke (Camel unfiltered, R.J. Reynolds Co.) through sterile saline, as described previously [24, 29]. The final CSE solution was adjusted to pH 7.2–7.4 and was prepared each day immediately before experimental testing to minimize potential degradation of the constituents. CSE was regularly analyzed by GC-MS (UCI Mass Spectrometry Facility), with some samples also sent to an outside facility (UCSF Clinical Pharmacology Laboratory), to verify nicotine content.

### Surgery

Adults (P85–87) and adolescents (P26–28) were anesthetized with equithesin (0.0035 mL/g body weight) and implanted with indwelling jugular vein catheters based on previously published methods [24]. Animals were anesthetized with Equithesin (0.3 mL/100 g, i.p. for adults, 0.25 mL/100 g, i.p. for adolescents) and a chronic catheter was passed subcutaneously from the animal’s back to the jugular vein where the tubing was inserted. Wounds were closed with clips, antiseptic ointment applied, and Baytril (0.1 mL/150 g, i.m.) was injected to prevent infection. During the 2–3 days recovery period, and for the remainder of the study, animals were flushed daily with heparinized saline solution (1 mL of 1,000 units/mL heparin into 30 mL bacteriostatic saline).

### Chronic drug treatment

Following recovery from surgery, adult (P89-91) and adolescent rats (P30-32) were weighed daily and given passive intravenous injections of saline, nicotine or CSE in an operant chamber programmed to deliver one injection per minute for 15 min (15 infusions per session), to yield a total of 0.5 mg/kg nicotine (free base) or CSE nicotine content per session [total of 0.5 mg/kg nicotine (free base) or CSE nicotine content per session (33.33 μg/kg nicotine content/infusion; 70 μL/infusion in 3.92 s for adults and 35 μL/infusion in 1.96 s for adolescents)]. Rats received three daily infusion sessions (9 a.m., 12 p.m., 3 p.m.) totaling 1.5 mg/kg/day of nicotine content for 10 consecutive days, including weekends. The dose of nicotine delivered is within the range of prior self-administration studies with nicotine and CSE in adolescents and adults [30].

### Spontaneous somatic withdrawal

For spontaneous somatic withdrawal, rats underwent withdrawal scoring before surgery and drug treatment began, and 1, 4, 18, and 48 h after the last drug injection. Somatic symptoms were assessed for 30 min following 30 min habituation to the open field chamber (17″ × 17″ × 12″) (Med Associates, St. Albans, VT). An observer blind to drug groups scored the following symptoms: body shakes, tremors, eye blinks, genital licks, gasps, head shakes, ptosis, teeth chattering, yawns, and writhes [30]. Withdrawal was defined as a significant increase in total withdrawal symptoms as compared to the saline group at the same time point. Catheter patency was verified by rapid anesthesia following infusion of 0.1 mL of propofol (Abbott Laboratories, Chicago, IL) after scoring the 4 h withdrawal time point. Animals without patent catheters were excluded from analysis.

### Precipitated somatic withdrawal

To investigate nAChR involvement in CSE withdrawal, a separate group of animals received an injection of saline or mecamylamine (1 mg/kg. s.c.), a non-selective nAChR antagonist, immediately following the last drug infusion, and were placed in the open field chamber and scored for somatic withdrawal symptoms for 60 min. Withdrawal was defined as a significant increase in total withdrawal symptoms as compared to the vehicle treated group. Catheter patency was verified for rapid anesthesia by infusing 0.1 mL of propofol (Abbott Laboratories, Chicago, IL) immediately following the test. Animals without patent catheters were excluded from analysis.

### Spontaneous affective withdrawal

Spontaneous affective withdrawal was measured 18 h following the last drug infusion using the light-dark box test for anxiety-like behavior. Animals were isolated in a plexiglass cage (16″ × 16″ × 12″) in the behavior testing room for 10 min, then were placed in the dark side of a light-dark box (17″ × 8.5″ × 12″ each side) (Med Associates, St. Albans, VT) and the time spent in the light versus dark chambers was recorded for 5 min [31]. Anxiety-like behavior was defined as an increase in the time spent in the dark side as compared to the saline group. The same groups of animals were tested for anxiety-like behavior using analysis of center time in an open field 30 days following the last drug infusion. Animals were isolated in a plexiglass cage in the behavior testing room for 10 min, then were placed in the center of an open-field chamber (43.2 cm × 43.2 cm × 30.5 cm) and the time spent in the center versus the periphery was recorded for 5 min. Anxiety-like behavior was defined as a decrease in the time spent in the center of the open field chamber as compared to the saline group.

### Precipitated affective withdrawal

To measure precipitated affective withdrawal, animals were injected with saline or mecamylamine (1 mg/kg, s.c.) immediately following the last drug infusion and isolated in a plexiglass cage for 20 min. After isolation, and a 5 min room habituation, the rats were placed in the dark side of a light-dark box (Med Associates, CA) and the time spent in the dark versus light chambers was recorded for 5 min. Anxiety-like behavior was defined as an increase in the time spent in the dark compartment as compared to vehicle treated groups.

### Data analysis

Age differences in mean total spontaneous somatic withdrawal symptoms following chronic nicotine or CSE treatment were analyzed with a 3-way ANOVA for Age × Drug × Time, with repeated measures on Time. Significant main effects were analyzed further with ANOVAs and Bonferroni-corrected paired or unpaired t-tests, where appropriate. For spontaneous affective withdrawal, the % time spent in the dark side and the % time spent in the center were analyzed with a 2-way ANOVA for Age x Drug. Significant main effects were analyzed further with Bonferroni-corrected unpaired t-tests. For precipitated withdrawal, differences in mean total precipitated somatic withdrawal symptoms and % time spent in the dark side were analyzed with a 3-way ANOVA for Age x Drug x Antagonist dose. Significant main effects were analyzed further with ANOVAs and unpaired t-tests.

## Results

### Spontaneous somatic withdrawal

**FIGURE 1:**
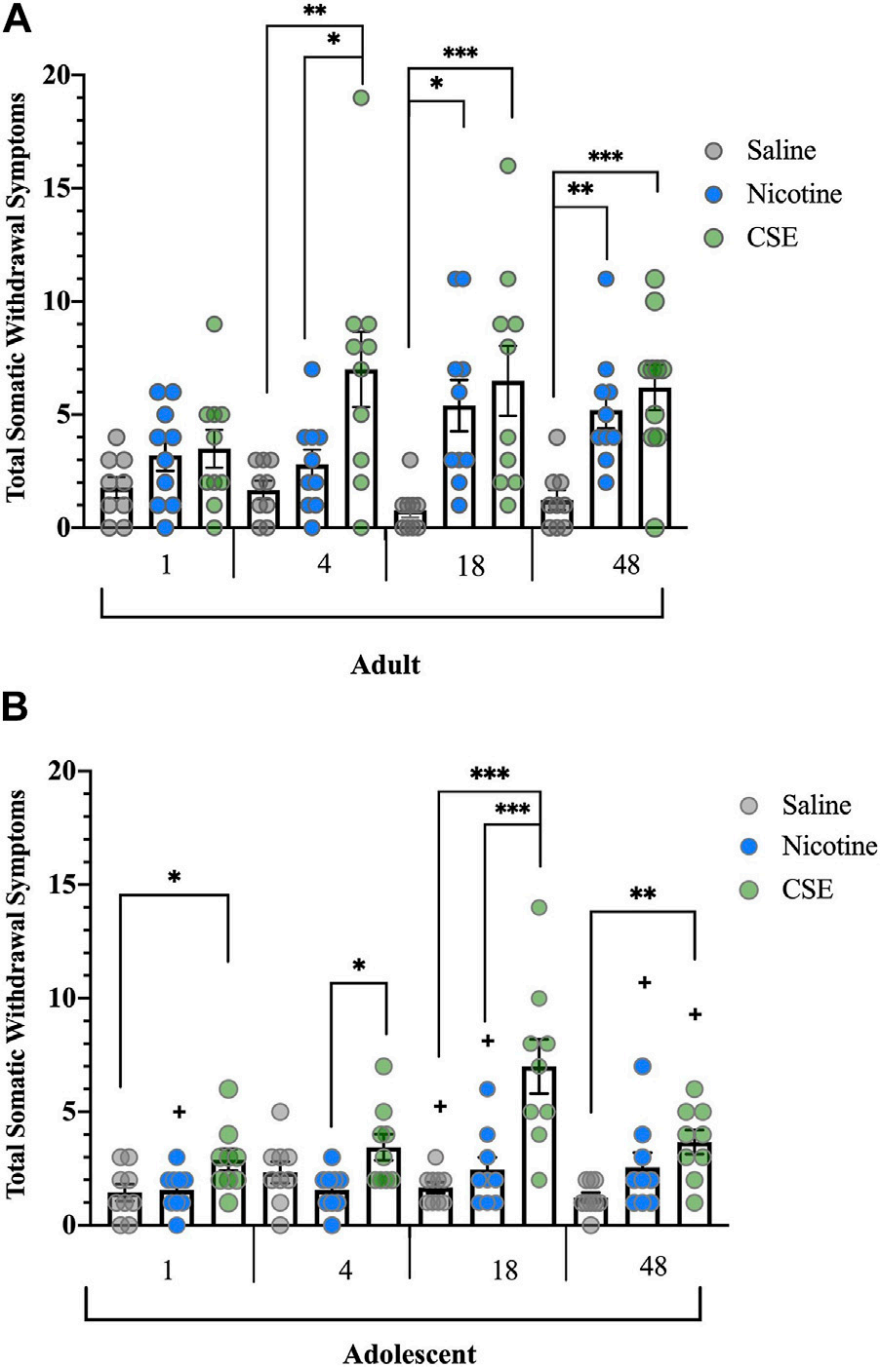
Spontaneous somatic withdrawal in adult and adolescent rats. **(A)** Withdrawal from CSE in adult rats emerges sooner and is more severe than from nicotine alone. **(B)** Adolescent rats withdraw after cessation from chronic CSE treatment but not chronic nicotine treatment. Animals were scored for somatic abstinence signs at various time points after last drug treatment. *p ≤ 0.05, **p ≤ 0.01, ***p ≤ 0.001; ^**+**^ *p* ≤ 0.05 vs. adults. *n* adults = 9–10, *n* adolescents = 9 per group.

3-way ANOVA revealed overall effects of Age (F_1,50_ = 8.185, *p* = 0.006), Drug (F_2,50_ = 26.626, *p* = 0.000), Time (F_3,150_ = 4.698, *p* = 0.004), with Age × Drug (F_2,50_ = 3.436, *p* = 0.04) and Time × Drug (F_6,150_ = 3.508, *p* = 0.002) interactions. Adults treated with CSE showed significant spontaneous somatic signs earlier than those treated with nicotine (Figure 1A). Overall ANOVA showed significant effects of Drug (F_2,26_ = 14.894, *p* = 0.001). CSE withdrawal, defined as significantly different from saline-treated controls, was evident 4 h after the last CSE infusion (*p* = 0.006) and was significantly greater than that of rats treated with nicotine alone (*p* = 0.03). Significant nicotine and CSE withdrawal were seen 18 h (*p* = 0.029, *p* = 0.006, respectively), and at 48 h (*p* = 0.005, *p* = 0.001, respectively) after the last drug infusion.

Spontaneous cessation of CSE, but not nicotine, resulted in somatic withdrawal in adolescent rats (Figure 1B). There were significant Drug (F_2,24_ = 17.041, *p* < 0.001) and Time effects (F_3,72_ = 6.589, *p* = 0.001), and a significant Time × Drug interaction (F_6,72_ = 4.674, *p* < 0.001). As has been reported previously [18, 19], adolescent rats did not show somatic withdrawal signs after a moderate dose or schedule of chronic nicotine treatment at any time point. Adolescent animals treated with CSE showed significantly higher somatic withdrawal signs than those treated with saline at 1, 18, and 48 h (*p* = 0.047, *p* < 0.001, *p* = 0.006, respectively) or nicotine at 4 and 18 h (*p* = 0.025, *p* = 0.001).

Adults treated with saline showed higher somatic signs than adolescents at 18 h (F_1,16_ = 4.923, *p* = 0.041). Adults treated with nicotine showed higher somatic signs than adolescents at 1 h (F_1,17_ = 4.554, *p* = 0.048), 18 h (F_1,17_ = 5.074, *p* = 0.038), and 48 h (F_1,17_ = 6.412, *p* = 0.021). Adults treated with CSE showed higher somatic signs than adolescents at 48 h (F_1,17_ = 4.715, *p* = 0.044) but not at earlier time points.

### Precipitated somatic withdrawal

**FIGURE 2:**
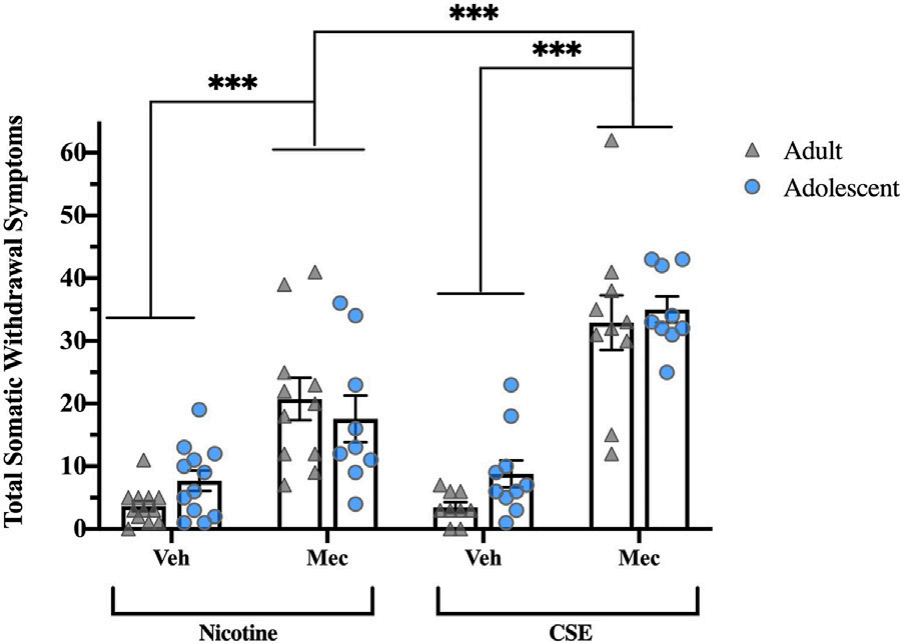
Precipitated somatic withdrawal in rats treated with CSE is greater than rats treated with nicotine alone. Animals were given vehicle or mecamylamine (1 mg/kg, s.c.) following drug treatment and scored for somatic signs for 60 min ***p ≤ 0.001. *n* = 6–11 per group.

Precipitated somatic withdrawal was higher in both adolescents and adults chronically treated with CSE than those treated with nicotine (Figure 2). Overall ANOVA showed significant Drug (F_1,74_ = 16.819, *p* < 0.001) and Pretreatment effects (F_1,74_ = 123.144, *p* < 0.001), with a significant Drug × Pretreatment interaction (F_1,74_ = 14.871, *p* < 0.001). No significant Age effect or interaction was observed, in contrast to spontaneous withdrawal. Whereas injection with mecamylamine (1 mg/kg, s.c.) increased somatic withdrawal signs in animals treated with CSE or nicotine (*p* < 0.001). precipitated withdrawal signs were significantly higher in those treated with CSE (*p* < 0.001).

### Spontaneous anxiety-like behavior

**FIGURE 3:**
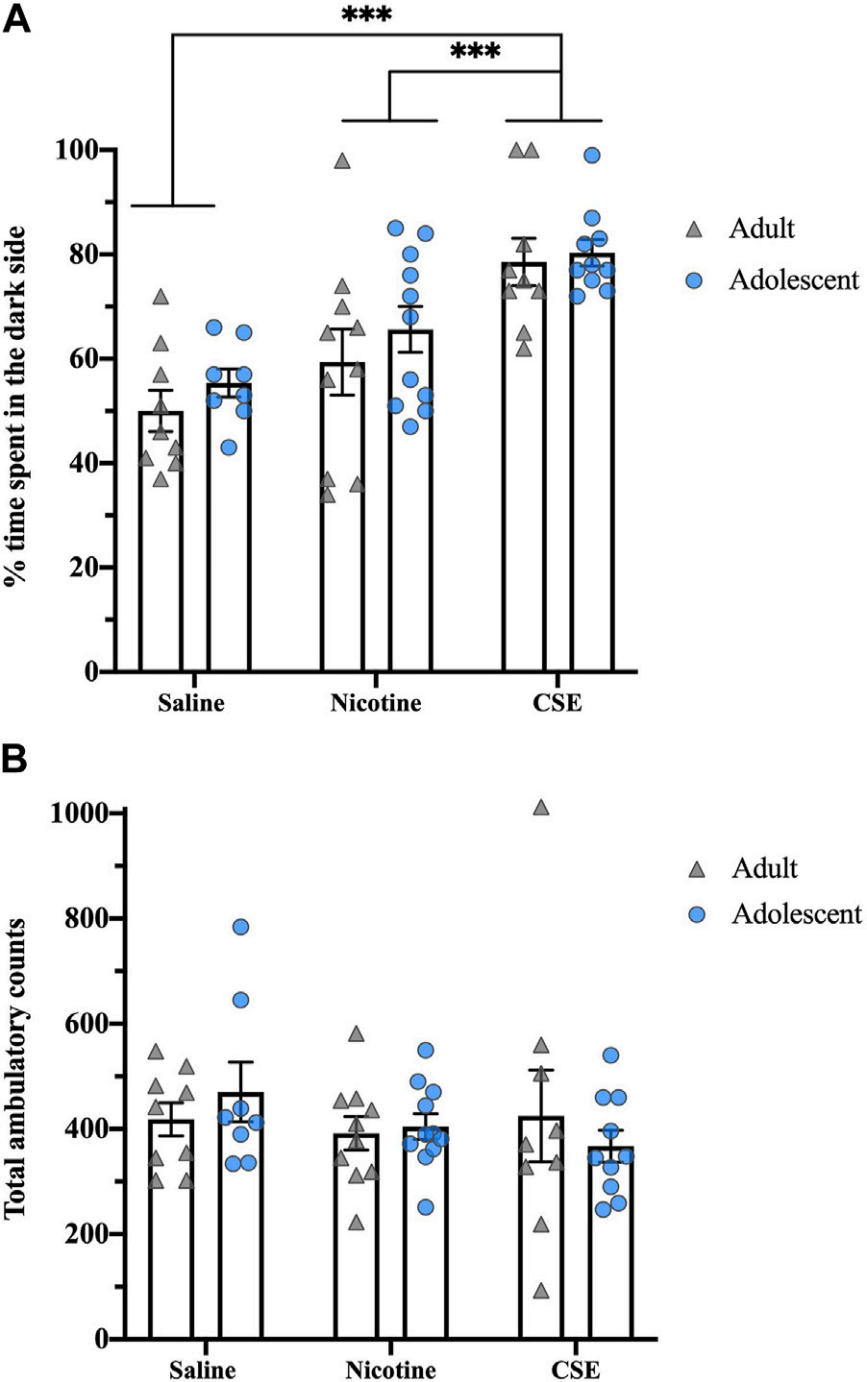
Drug, but not age, differences in anxiety-like behavior in a light-dark box test at 18 h post drug treatment. **(A)** The percent of time spent in the dark side of the light-dark box was recorded for 5 min. **(B)** Total ambulatory counts were recorded as a measure of locomotion. ***p ≤ 0.001. *n* = 8–11 per group.

Eighteen hours after the last drug infusion, CSE-treated rats showed greater affective withdrawal signs than those treated with nicotine (Figure 3A). Although there was a significant effect of Drug (F_1,51_ = 18.281, *p* < 0.001), there was no Age or Age × Drug interaction. Chronic CSE treatment significantly increased the time animals spent in the dark compartment as compared to chronic treatment with saline (*p* < 0.001) or nicotine (*p* = 0.001). There were no significant differences in total ambulatory counts, showing that the anxiety-related behavior did not result from locomotor effects (Figure 3B).

**FIGURE 4:**
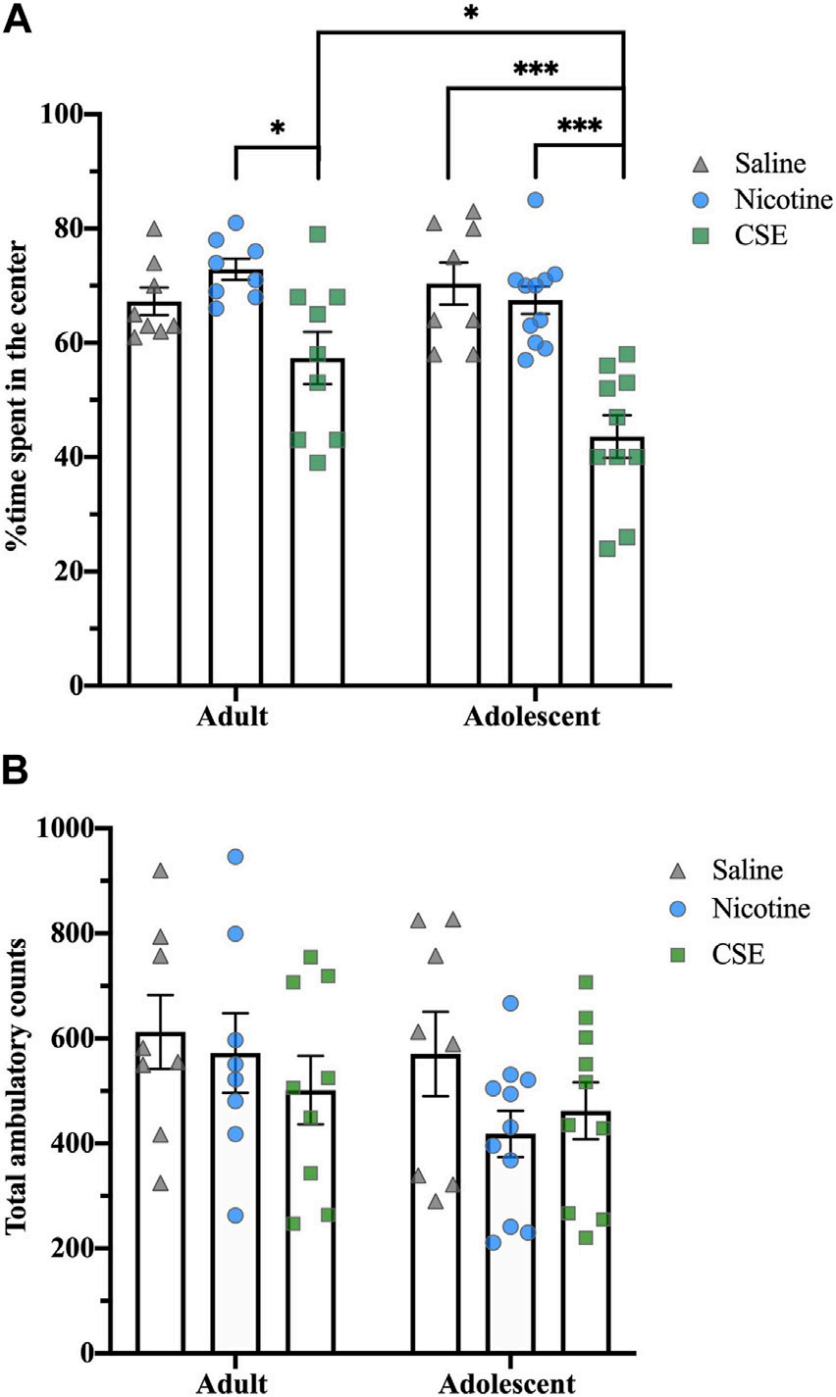
Age and drug differences in anxiety-like behavior in an open field at 30 days post treatment. **(A)** The time spent in the center of the open field box was recorded for 5 min. **(B)** Total ambulatory counts were recorded as a measure of locomotion. *p ≤ 0.05; ***p ≤ 0.001. *n* = 8–11 per group.

Thirty days after cessation of drug treatments, animals still showed anxiety-like behaviors, as measured by time spent in the center of an open field (Figure 4A). At this time, there was an overall effect of Drug (F_2,48_ = 5.724, *p* < 0.001) with a strong trend for an Age effect (F_1,48_ = 3.876, *p* = 0.055) and Age × Drug interaction (F_2,48_ = 3.171, *p* = 0.051). Adults showed an overall effect of Drug (F_2,22_ = 23.021, *p* = 0.01), with CSE-treated rats spending less time in the center of the field than those treated with nicotine (*p* = 0.009) but not saline. Adolescents showed an overall Drug effect (F_2,26_ = 20.694, *p* < 0.001), with CSE-treated rats spending less time in the center of the field than saline- or nicotine-treated animals (*p* < 0.001). Adolescents treated with CSE also spent significantly less time in the center field than adults treated with CSE (*p* = 0.032), showing that adolescents are more susceptible to long-term anxiogenic effects of chronic CSE treatment than adults. The anxiety-like behaviors following long-term drug withdrawal did not result from locomotor effects, as there were no overall Drug or Age effects for total ambulatory counts (Figure 4B).

### Precipitated anxiety-like behavior

**FIGURE 5:**
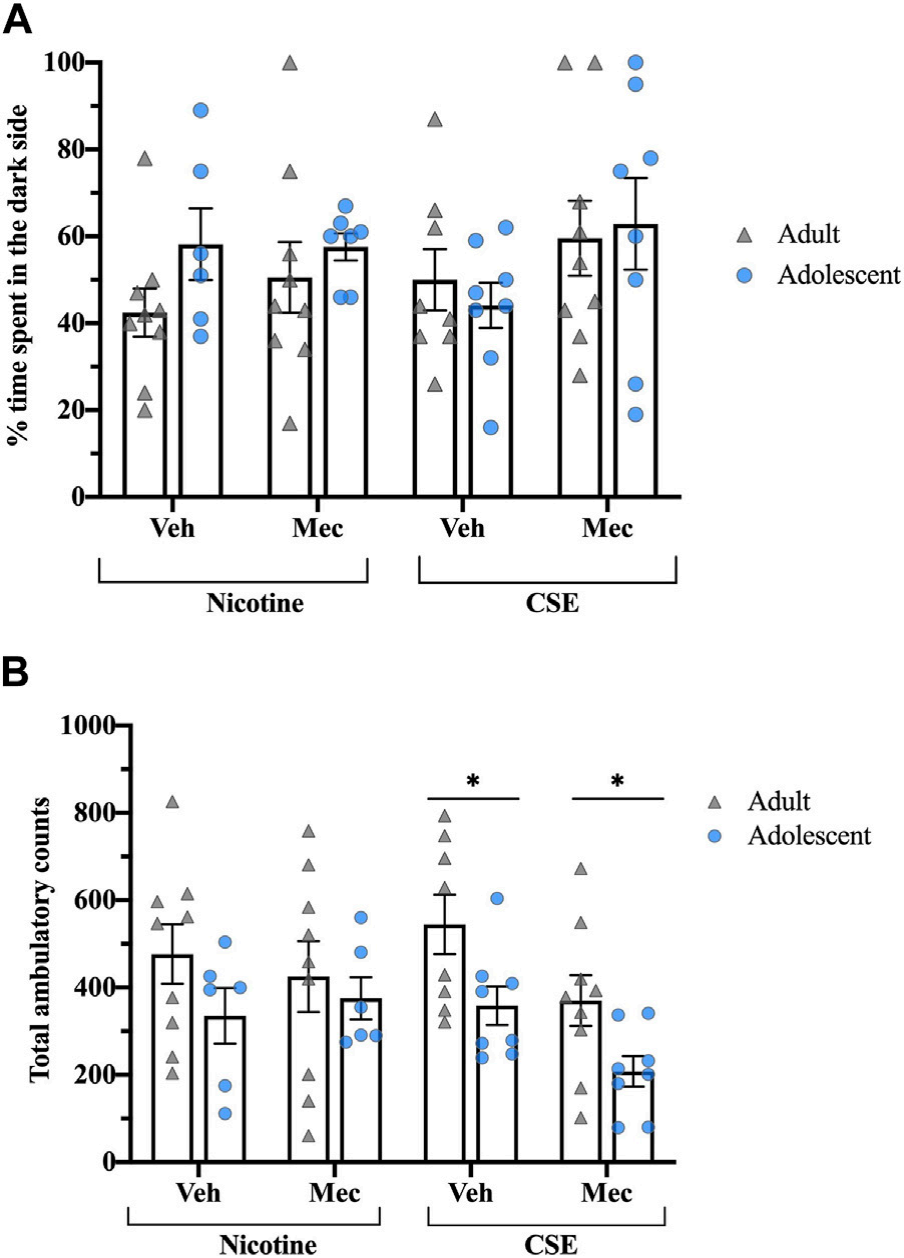
Rats treated with CSE or nicotine show no differences in precipitated affective withdrawal. **(A)** The time spent in the dark side of the light-dark box test was recorded for 5 min following a saline or mecamylamine injection (1 mg/kg, s.c.). **(B)** Total ambulatory counts were recorded as a measure of locomotion. *p ≤ 0.05 adult vs. adolescent. *n* = 6–9 per group.

Mecamylamine did not precipitate anxiety-like behavior in rats treated with CSE or nicotine (Figure 5A). Total ambulatory counts showed a significant effect of Age (F_1,55_ = 9.139, *p* = 0.004), with adolescents treated with CSE moving less than adults (*p* < 0.05) (Figure 5B).

## Discussion

The current study shows that CSE enhances spontaneous somatic and affective withdrawal in adult and adolescent rats as compared to chronic treatment with nicotine alone [28]. Since there was equivalent nicotine content across drug groups, this indicates that the non-nicotinic constituents in CSE contribute to neuroadaptations that occur during the formation of dependence, resulting in a greater withdrawal syndrome upon cessation. Animals treated with CSE showed higher mecamylamine-precipitated somatic withdrawal than animals treated with nicotine alone, suggesting that cigarette smoke constituents may enhance somatic withdrawal via a nAChR-based mechanism. Whereas termination of CSE treatment increased anxiety as compared to treatment with nicotine alone, the role of nAChRs is less clear, since mecamylamine did not precipitate affective withdrawal following either CSE or nicotine treatment.

### Methodological issues

The study of tobacco use with preclinical models has been challenging as it is difficult to find an appropriate method to treat animals in a way that best mimics human smoking [28]. Although studies with smoke inhalation have demonstrated withdrawal after chronic treatment in adult and adolescent rats, these studies best model passive tobacco smoke inhalation [21, 32]. Another common way to chronically treat animals with nicotine is with an osmotic pump which maintains constant infusions over a chronic period [15–19]. This method does not model the daily perturbations of tobacco use in smokers, however. Nor does it permit alterations in the level of drug delivery as a developing animal grows. The treatment paradigm in the present study was via passive intravenous infusion, an approach that allowed daily preparation of CSE, which has non-nicotinic constituents of unknown stability. A further advantage of this intravenous administration protocol is that it allows easy control of the dose of nicotine that animals receive daily, which is particularly important in adolescent animals that are experiencing a rapid growth spurt. Thus, in contrast to earlier studies [18, 19, 33], the current infusion methodology allows direct comparison of the effects of equivalent chronic drug doses in adolescent and adult rats. A prior study in adolescent mice has shown that similar behavioral results can be obtained following chronic nicotine administration via osmotic minipumps or daily injections [34].

### Nicotine withdrawal

Adolescent rodents have been shown previously to be less sensitive than adults to the effects of nicotine withdrawal [18, 19, 34]. This contrasts clinical studies that show adolescents have greater withdrawal than adults when quitting smoking [7, 8, 35]. We have replicated the finding that somatic symptoms following withdrawal of chronic nicotine treatment are lower in adolescent rats than adults. In contrast, we found no age differences in somatic symptoms when withdrawal was precipitated with mecamylamine. This contrasts with other reports in which mecamylamine-precipitated somatic withdrawal signs were less intense in adolescent rodents than adults [19, 34].

Neither adolescent nor adult rats showed significant withdrawal-induced anxiety either 18 h or 30 days following cessation of nicotine treatment, nor did mecamylamine precipitate anxiety-like behavior. These findings contrast with earlier reports; however, those studies used higher doses of nicotine [34, 36, 37].

### CSE withdrawal

Withdrawal symptoms in both adolescent and adult rats were significantly more pronounced following chronic treatment with CSE than with nicotine. In contrast to nicotine-exposed adolescents who exhibited no somatic signs, spontaneous withdrawal from CSE produced adolescent somatic symptoms of similar intensity to those seen in adults. In contrast, no age difference was seen in mecamylamine-precipitated somatic signs in chronically CSE treated rats.

Eighteen hours following spontaneous withdrawal from CSE, both age groups showed enhanced anxiety-like behavior in a light-dark box test as compared to nicotine-treated rats or saline controls. Anxiety-like behavior in an open field test was still evident 30 days after spontaneous CSE withdrawal and was greater in adolescents than adults. Thus, the withdrawal syndrome that adolescents experience following chronic CSE exposure may be different from that in adults, with greater affective than somatic symptoms and a different time course. Similar long-term elevation in anxiety-like behavior has been seen in adult male mice following chronic inhalation exposure to cigarette smoke [32], and in adolescent rats given chronic daily nicotine injections [38, 39].

Although chronic treatment with CSE increased spontaneous anxiety-like behavior in adult and adolescent rats, mecamylamine did not precipitate affective withdrawal in either age group. This contrasts with what was observed for somatic withdrawal symptoms. There are several other reports that spontaneous and mecamylamine-induced withdrawal may not yield identical effects on the same behavioral test [15, 19, 40, 41]. The reason for this is not clear but may reflect the differential affinity of mecamylamine for multiple nAChRs [42].

### Nicotinic receptors (nAChRs)

Prior studies have shown that different nAChRs have prominent roles in somatic and affective withdrawal symptoms. Mice with null mutation of β4 or α5 nAChRs subunits have fewer somatic signs of withdrawal [43, 44]. In contrast, elimination of β2 or α6 nAChR subunits attenuates affective withdrawal symptoms [43, 45]. We have previously shown that rats undergoing a chronic CSE treatment procedure identical to that used in the current study exhibit changes in nAChR binding in various brain regions 1 h after the last drug infusion [46]. Daily nicotine treatment did not significantly increase nAChR binding in any brain region at either age, a finding that contrasts with prior studies using higher drug doses and/or more chronic treatments [47–49]. Daily CSE exposure selectively increased adolescent α4β2 nAChR binding in medial amygdala and α7 nAChR binding in central amygdala and lateral hypothalamus. CSE also increased α3β4 nAChR binding in the medial habenula and interpeduncular nucleus, and α7 binding in the medial amygdala, independent of age. Almost all the brain regions in which CSE induced significant changes in nAChR binding have been associated with nicotine withdrawal behavior [50, 51]. The medial habenula—interpeduncular nucleus pathway is particularly implicated in both somatic and affective signs of nicotine withdrawal [52–54]. Interpeduncular nucleus GABAergic neurons are dynamically regulated during nicotine withdrawal, with resulting anxiety-like and somatic symptoms [55]. Furthermore, infusion of a β4-selective nAChR antagonist into the interpeduncular nucleus elicits somatic signs of nicotine withdrawal [56]. Thus, the CSE-induced increases in α3β4 nAChR binding seen in the medial habenula—interpeduncular nucleus pathway at both ages may underlie the observed increases in somatic withdrawal signs. Although the animals were currently exposed to CSE by passive exposure, it should be noted that we have previously noted that adult rats who self-administered CSE exhibit functional changes in α3β4 nAChRs mediating reinstatement of drug-seeking behavior [57].

### Non-nicotine constituents

Although substantial work has been done on the identity of non-nicotine constituents that impact nicotine reinforcement and reward [24, 26, 58–62], there are few studies that have looked at the impact of individual constituents on nicotine withdrawal. One exception is monoamine oxidase inhibitors (MAOIs) in tobacco which reduce enzyme activity in the brains of smokers [63] and are present in CSE extracts [24, 64]. In rats, chronic MAOI treatment induces a prolonged conditioned placed aversion associated with nicotine withdrawal [25]. Furthermore, acute inhibition of MAO-A induces significantly greater nicotine somatic withdrawal signs than in control rats [26]. Thus, it may be MAOIs within CSE that induce greater somatic and affective withdrawal symptoms observed in the current study. Further experiments are required to examine the impact of MAOI inhibition on nicotine withdrawal in adolescents, and to determine whether there are similar alterations in α3β4 nAChRs observed in chronic CSE studies [40, 57].

### Limitations

The current study has a few limitations. One of the major challenges in studying smoking in animals is using a model that best models smoking in humans, with use of CSE being no exception. In this study we used three times daily intravenous injections of drugs rather than the more commonly used osmotic minipump. There were advantages to this approach, including mimicking human consumption patterns, regular refreshing of CSE solution and matching drug intake to body weight in growing adolescents. However, given this different route of administration, we cannot fully compare our findings with those of earlier studies. Furthermore, although adolescent and adult animals were given matching doses, blood levels of nicotine and cotinine have been found to be lower than adults, impacting the validity of age comparisons [65]. Another limitation is that CSE contains only the aqueous constituents of cigarette smoke, and not the remaining ∼60% of non-aqueous components [28, 66]. The extracts commonly used in tobacco research are prepared in an organic solvent in order to dissolve the tar phase of the smoke [67, 68]. Since the current experimental paradigm requires intravenous infusion of CSE, an organic solvent was not practical.

One final limitation is the absence of female animals in our experimental design. As with the current study, most prior studies of nicotine withdrawal have focused on male rodents. However, substantial sex differences have been observed in both clinical and preclinical studies. Adult women are more likely than men to relapse after quitting cigarette use because of withdrawal symptoms, including tension, anxiety and craving [69, 70]. Adolescent females also report higher levels of stress and depression during withdrawal than their male counterparts [71]. Preclinical studies have similarly shown that adult, but not adolescent, females exhibit greater somatic and affective withdrawal symptoms than males [36, 72]. Enhanced withdrawal-induced anxiety in females is associated with greater corticosterone and ACTH release than males, indicating a more activated stress response [36, 73]. A recent study has shown that sex differences in glutamate and GABA response in the interpeduncular nucleus may mediate behavioral differences in withdrawal [74]. Given our current findings that CSE induces greater somatic and affective withdrawal signs in both adult and adolescent males as compared to nicotine alone, it will be very important to repeat this study in females.

### Conclusion

Clinical studies in adults have indicated that smoking cigarettes produces higher levels of dependence than vaping e-cigarettes [12]. Similarly, adult mice chronically exposed to cigarette smoke show greater somatic and affective symptoms of withdrawal than those exposed to e-cigarette vapor [75]. In the present study, we have confirmed that non-nicotine tobacco constituents in CSE administered intravenously enhance both physical and affective symptoms of nicotine withdrawal in adult rats. We have previously shown that these constituents do not impact nicotine self-administration, but do enhance reinstatement induced by the pharmacological stressor, yohimbine [24, 27] and elevate and increase intracranial self-stimulation thresholds to a greater degree than high doses of nicotine alone [64]. Thus, tobacco constituents appear to increase the negative impact of withdrawal from chronic nicotine.

Given the recent substantial increase in e-cigarette use by teenagers [4–6], it is important to evaluate whether this mode of nicotine delivery produces similar levels of dependence to that of tobacco cigarettes. To date there have been no direct comparisons of the effect of tobacco smoke constituents. We have confirmed that chronic nicotine treatment results in higher somatic withdrawal signs in adolescents than adults. We have also shown that chronic nicotine, at this low dose, does not result in different anxiety-like behaviors in adolescents and adults following acute and long-term withdrawal. We now show that chronic CSE induces greater levels of both physical and affective dependence in adolescent rats than equivalent doses of nicotine alone. Our findings agree with prior studies that nicotine alone produces very little physical dependence in adolescent rodents [18, 19]. However, tobacco constituents increase somatic withdrawal symptoms in adolescents to levels observed in adults. Adolescents also show similar anxiety to adults immediately following spontaneous withdrawal of CSE and continue to exhibit this 1 month later at a level greater than that of adults. These findings complement our earlier observations that cigarette smoke constituents enhance yohimbine-induced reinstatement of nicotine seeking behavior in adolescents [27]. Future studies will be required to determine the mechanism of CSE action in enhancing withdrawal signs. However, it seems likely that elevated levels of α3β4 nAChR binding in the habenulo-peduncular pathway underlies the increased somatic symptoms of withdrawal in both adolescents and adults. Overall, our findings indicate the importance of using CSE, instead of nicotine alone, in preclinical models of tobacco dependence.

## Data Availability

The raw data supporting the conclusion of this article will be made available by the authors, without undue reservation.
